# Programming of Cell Resistance to Genotoxic and Oxidative Stress

**DOI:** 10.3390/biomedicines6010005

**Published:** 2018-01-02

**Authors:** Ilya O. Velegzhaninov, Vitaly A. Ievlev, Yana I. Pylina, Dmitry M. Shadrin, Olesya M. Vakhrusheva

**Affiliations:** 1 Institute of Biology of Komi Science Centre of Ural Branch of RAS, Syktyvkar 167982, Russia; yanapylina@yandex.ru (Y.I.P.); shdimas@yandex.ru (D.M.S.); 2 Polytechnical Institute of Vyatka State University, Kirov 610000, Russia; nimf04ka@mail.ru; 3 Biology department of St. Olaf College, Northfield, MN 55057, USA; ievlev1@stolaf.edu

**Keywords:** cell programming, stress resistance, gene overexpression, radiation, oxidative stress, chemical genotoxins, malignant transformation, diversity of mechanisms

## Abstract

Different organisms, cell types, and even similar cell lines can dramatically differ in resistance to genotoxic stress. This testifies to the wide opportunities for genetic and epigenetic regulation of stress resistance. These opportunities could be used to increase the effectiveness of cancer therapy, develop new varieties of plants and animals, and search for new pharmacological targets to enhance human radioresistance, which can be used for manned deep space expeditions. Based on the comparison of transcriptomic studies in cancer cells, in this review, we propose that there is a high diversity of genetic mechanisms of development of genotoxic stress resistance. This review focused on possibilities and limitations of the regulation of the resistance of normal cells and whole organisms to genotoxic and oxidative stress by the overexpressing of stress-response genes. Moreover, the existing experimental data on the effect of such overexpression on the resistance of cells and organisms to various genotoxic agents has been analyzed and systematized. We suggest that the recent advances in the development of multiplex and highly customizable gene overexpression technology that utilizes the mutant Cas9 protein and the abundance of available data on gene functions and their signal networks open new opportunities for research in this field.

## 1. Introduction

Genotoxic stress, including oxidative stress, causes DNA damage. The evolutionary conservative cellular mechanisms of DNA-damage prevention and response (DNA repair, defense against reactive oxygen species, cell cycle checkpoints, and apoptosis) protect cells from mutations and tissues from acquiring malignancy [1,2]. On the one hand, genotoxic stress can induce carcinogenesis, on the other hand, it is used to treat cancer. The advancement of knowledge on regulation of stress-resistance in cells and organisms is extremely important for increasing the effectiveness of cancer treatment. In particular, the creation of new in vitro models of upregulated cell resistance to genotoxic and oxidative stresses allows for expanding the spectrum of in vivo models for studies of genetic regulation of carcinogenesis. In addition, it was suggested multiple times that gene therapy of normal tissues surrounding tumor can be used for increasing their resistance to genotoxins. This can help to minimize the negative side effects of cancer treatment by chemotherapy and radiation therapy [3,4,5]. This technology can also be used for gene therapy and gene prophylaxis of diseases that are associated with increased sensitivity to DNA-damaging agents [6]. Understanding the mechanisms of cellular stress resistance, and especially resistance to oxidative stress, is one of the most important tasks in studies of lifespan extension [7,8]. Knowledge of stress-resistance is also important when creating new genetically modified varieties of plants and breeds of animals [9]. Additionally, the problem of prolonged exposure of astronauts to cosmic ionizing radiation is a great challenge that needs to be addressed in order to make deep space expeditions possible [10,11]. One of the possible solutions is a pharmacological or genotherapeutic enhancement of human radioresistance. Lastly, cell cultures with multiple enhanced stress resistance can find application in recombinant therapeutic protein production [12]. To achieve all of the objectives listed, except for the last one, it is necessary to ensure that tissue function and cells’ ability to elicit apoptotic and cell cycle responses are both not affected as a result of genetic engineering interventions. Ideally, an increase in resistance to genotoxic stress should lead to a decrease in the frequency of somatic mutations and neotransformations at the organismal level.

The functions of many stress-response genes have been well studied. Signal-cascade networks of gene activation in response to various damaging agents have also been elucidated. Such knowledge can help in identifying potential gene targets and their combinations for transcriptional activation increasing resistance to genоtoxic and oxidative stress. However, without an array of experimental data, it is not possible to accurately predict the results of such activations. Moreover, it is difficult to prognosticate the biological consequences of varying degree overexpressions of one and the same gene. The discovery of the CRISPR/Cas adaptive immunity and the development of methods for its application for genome [13] and epigenome editing [14,15,16,17,18] significantly expands the possibilities for further studies of stress resistance programming. In particular, relatively simple and adjustable multiplex overexpression of genes by nuclease-null Cas9 (dCas9) can successfully activate multi-subunit molecular complexes or entire signal cascades. Moreover, this technology provides the activation of genes in endogenous context, covering splice variants [16]. These advantages distinguish it from the previously dominant gene overexpression technology, which relies on introduction of cDNA into the cell under a constantly active or inducible promoter. To date, there are very few works in the literature that used CRISP/dCas9 for gene overexpression. Most of the articles are devoted to the optimization of the technology and its application in various fields of biological science. However, this technology has already begun to prove its high potential. For example, it was shown the possibilities of reactivation of silenced tumor supressors in vitro [19] and the regulation of tumor phenotypes in vivo [20]. Thus, we expect a new round of research in the field of genetic and epigenetic regulation of resistance to genotoxic stress. In this regard, the review discusses the current state of knowledge about modulation of resistance to genotoxic and oxidative stress by genes overexpression in case of normal and cancer cells, as well as whole organisms. To assess the potential of genetic regulation of stress-resistance the review also discusses transcriptomic studies in cancer cells with different levels of radioresistance.

## 2. The Diversity of Mechanisms of Stress Resistance in Cancer Cells

Mechanisms for development of genotoxic and oxidative stress resistance in tumor cells are well described in a number of reviews [21,22,23,24]. Clearly, cells lacking the capacity for apoptosis or irreversible cell cycle arrest will exhibit a resistant phenotype due to their continued ability to proliferate even under severe genotoxic stress conditions. Continued exposure to genotoxins in a combination with abnormal response to DNA damage can lead to a further loss of control mechanisms and can increase resistance to stress in tumor cells. For example, this can happen through the missense mutations in tumor suppressors. It can also be induced by a shift in a balance between homologous (HR) and non-homologous end-joining (NHEJ) in double strand break DNA repair [22]. In addition, resistance to genotoxic stress is associated with the activation of oncogenes *N-ras*, *K-ras* [25,26], *MET* [27], and *YAP* [28]. Radioresistance is also associated with the activity of the *Sox2* and *Oct3/4* genes that induce pluripotency and stem cell-like properties in cancer cells [29].

Due to the risk of carcinogenesis, the mechanisms described above cannot be used as practical targets for induction of cellular stress-resistance. However, stress resistance of tumor cells is often formed by the mechanisms that are not associated with initiation of malignant transformation. As mentioned above, alteration in components of genome stability machinery could lead to an increase in mutation rate in tumors, and result in an increased genetic heterogeneity of cells. This heterogeneity facilitates the rapid selection of cells subpopulations that are resistant to stress [23]. The possibility of this selection-based mechanism of resistance has been repeatedly confirmed in direct selection experiments [30,31,32]. However, there is also evidence that stress-resistance can be induced at the epigenetic level, independently from the selection process [33]. The resistance that is developed by selection or independently of it often results from the overexpression of the genes encoding transporter proteins, which support enhanced drug efflux [24]. In many cases, overactivation of DNA damage recognition and repair as well as detoxification of free radicals are also observed. For example, *Rad51* gene, which is involved in homologous recombination is overexpressed in a variety of human cancer types. This often leads to chemo-resistance of these tumors [34]. An inverse correlation was observed between the expression of the excision repair gene *ERCC1* and the sensitivity to platinum treatment of various types of tumors [35]. An enhancement of excision repair activity in lung cancer cells can also be associated with a SIRT1 dependent increase in XPA sensitivity to DNA damage [36]. Expression of the antioxidant defense gene—*MnSOD*—correlates with resistance to doxorubicin and mitomycin C in gastric carcinoma cells [37]. *RPA1* gene, which is involved in DNA replication and repair is overexpressed as a result of selection of a radioresistant clone in esophageal carcinoma cell line TE-1. Inhibition of RPA1 in that radioresistant clone restored the normal sensitivity to ionizing radiation [38].

**Figure 1:**
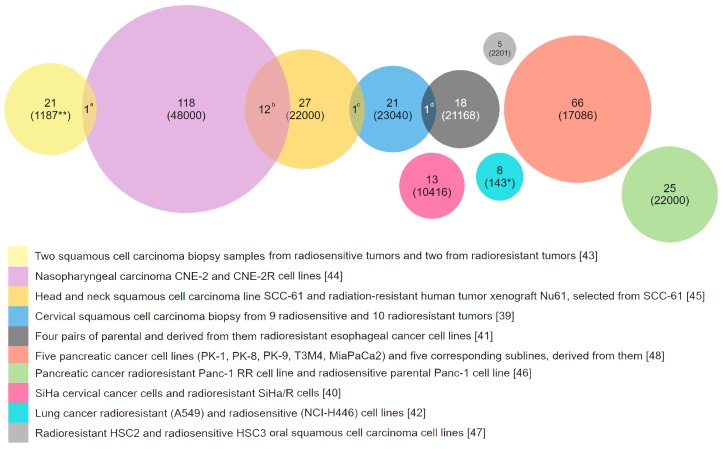
Genes that are overexpressed in radioresistant cancer cells in comparison with parental or similar but radiosensitive cells. The results of ten studies performed with microarrays were used. Only 15 of the 337 overexpressed genes are repeated twice in different studies: ^a^—*C-JUN*; ^b^—*CXCL10*, *IFI44*, *IFIH1*, *IFITM1*, *STAT1*, *DDX60*, *HERC6*, *IFI27*, *PLSCR1*, *IFIT1*, *IFI35*, *IFIT3*; ^c^—*ISG15*; ^d^—*ERP70*. Numbers in parenthesis is the quantity of transcripts analyzed. *—Genes that are involved in apoptosis, DNA repair, cell cycle control, cell proliferation and other mechanisms of stress response. **—only tumor-related genes.

There are many other examples of an established link between genotoxic stress resistance and overexpression of genes involved in DNA repair, xenobiotic detoxification, or efflux. However, the diversity of possible mechanisms of resistance seems to be even larger. This is supported by the studies comparing transcriptomes of similar cell lines that differ in sensitivity to genotoxic agents. For example, a comparison of ten microarray studies performed on cancer cells with different degrees of resistance to ionizing radiation did not identify any commonly overexpressed genes [39,40,41,42,43,44,45,46,47,48]. We could not find a gene that would be significantly overexpressed in three or more comparison pairs. Approximately 95% of the total number of overexpressed genes were observed in only one study and were absent in others (Figure 1). Interesting, that among the genes overexpressed in two different studies most are interferone induced genes, which involved in response to virus infection [49]. This fact shows once again that different systems can be involved in the regulation of resistance to genotoxic stress.

Thus, the diversity of pathways leading to resistance in cancer cells, allows for us to suggest a wide range of possibilities for increasing resistance of normal cells to genotoxic and oxidizing agents. We suppose, that if we exclude all of the targets that affect cell cycle control, apoptosis, proliferation, and differentiation, we can enhance stress-resistance without the risk of increasing malignancy. Moreover, the increased efficiency of cellular defense systems should in theory lead to a decrease in carcinogenesis. This assumption is supported by the fact that the activity of DNA repair systems inversely correlates with the risk of neotransformation [50]. In addition, a decrease in alkylating agent-induced carcinogenesis has been repeatedly demonstrated upon overexpression of the gene *O*^6^-methylguanine-DNA methyltransferase (*MGMT*), which is responsible for DNA damage recognition and repair [51,52,53,54,55,56].

## 3. Genotoxic Stress Resistance in Experimental Models with Gene Overexpression

Change in gene transcription is only one of the existing ways of readjusting the mechanisms of stress resistance. Another way of establishing stress resistance is a pharmacological targeting of proteins and signaling cascades, which seem more acceptable for clinical applications. However, accumulation of experimental data on the effects of overexpression of individual genes and their combinations is required to develop pathways of stress-resistance regulation that might help finding new pharmacological targets. The literature on the effects of overexpression of stress-responsive genes on the resistance of cells and organisms to genotoxins is overwhelmingly broad. However, we attempted to systematize such published experimental data based on overexpressed genes, on the effect on stress resistance and on genotoxicants. Being mindful of the scale and the variety of the published studies, in our analysis we chose a simple algorithm of grouping the target genes by their function. The resulting lists of reviewed published reports are presented in Table 1 and Table 2 for in vitro and in vivo studies, respectively. One interesting, but not totally surprising, finding of our analysis was that most studies driven by a targeted hypothesis (about involvement of a particular gene in stress resistance based on previous experimental evidence) found that overexpression of the gene did increase stress resistance. On the other hand, it seems that in case of randomly selected targets, the predominant outcome would be sensitization to stress, likely due to a disruption of normal gene activity regulation.

As suggested above, the two most promising gene categories to enhancing resistance by overexpression are the genes involved in DNA damage recognition and repair, as well as the genes that are responsible for efflux and detoxification of xenobiotics. Overexpression of these genes tends to be the most successful strategy of enhancing resistance to genotoxic stresses without the risk of increasing the frequency of neoplastic transformations. However, overexpression of these targets does not always lead to an expected/desired outcome. Firstly, an increase in survival can mask the decrease in DNA repair quality. For example, overexpression of the gene encoding DNA polymerase β in CHO cells lead to an increase in survival after treatment with cisplatin, melphalan, or mechlorethamine. However, it also dramatically increased the frequency of mutations in surviving cells. DNA polymerase β, the most error prone eukaryotic DNA polymerase [57,58,59,60] has been repeatedly shown to be the cause of the phenomenon mentioned Therefore, the required outcome and endpoints used should be selected carefully. Secondly, the effect of overexpression of various single elements of a repair or detoxification system/pathway can sometimes produce an effect that is opposite of the expected one. At the cellular level, the two main groups of reasons for this are (a) the imbalance between the elements of the protective systems; and, (b) the absence of the expected relationship between the level of gene transcription and the activity of the gene product. The latter primarily applies to all of the proteins whose activity depends on post-translational modifications. The mismatch between the mRNA levels and the protein function may also arise when a gene encodes only one subunit of multisubunit protein complexes. For example, stability of the DNA repair protein XPC depends on the levels of HR23A and HR23B proteins [61], therefore the overexpression of *XPC* gene may not be sufficient to enhance nucleotide excision repair. As consistent with this, an averaged quantitative relationship between the levels of mRNA and corresponding protein tends to be weak [62]. However, estimations of this correlation are still the subject of discussion and differ widely in the range from 0.21 to 0.9 [63]. In exceptional cases, for example, in the case of ribosomal proteins, mRNA can be a repressor of translation of its own product. This phenomenon is known to occur for the RpS3 protein that is involved in stress responses [64].

The imbalance of protective systems resulting from overexpression of individual genes may be caused by several different mechanisms. First, it can be driven by the imbalance in productivity of successive stages of a single cascade. For example, a wide range of modified bases in *S. cerevisiae* is excised using MAG1 (3-methyladenine DNA glycosylase). The abasic sites that are generated by MAG1 are processed normally by the major yeast APN1-encoded AP endonuclease. Disproportionately high expression of MAG1 when compared to the AP endonuclease increases spontaneous mutation by up to 600-fold in *S. cerevisiae* and by 200-fold in *E. coli* [65]. CHO cells with overexpressed *MPG* gene are more sensitive to alkylating agent *N*-methyl-*N*’-nitro-*N*-nitroso-guanidine (MNNG) that is also associated with excessive accumulation of abasic sites [66].

Secondly, there are situations when an increase in resistance to one agent is accompanied by sensitization to others. For example, overexpression of *APE1* increases the resistance of CHO cells to dioxolane cytidine [67], but it sensitizes cells to agents, which are activated by reduction reactions. This happens because the product of *APE1* gene has a RedOx function in addition to AP endonuclease activity [68]. Another mechanism is a shift in balance between the two competing processes. For example, the overexpression of *XRCC1* required for base excision repair (BER) slows gap-filling, because of the competition of BER with nucleotide excision repair for the PCNA protein [69].

**Figure 2:**
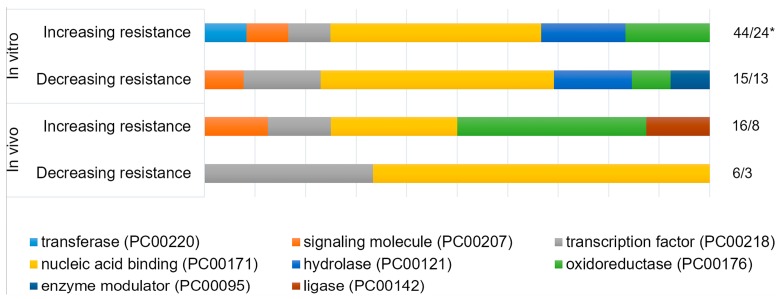
The functional classification of overexpressed genes using PANTHER classification system. Human orthologues of genes listed in Table 1 were divided into two groups, depending on the effect of their overexpression on the resistance of cells (“In vitro”). The same division was performed for orthologues of genes listed in Table 2 (“In vivo”). Each groups was classified using PANTHER Protein class ontology [70,71]. *—number of analyzed genes/total number of hits to “PANTHER protein class” classification.

The listed nuances of regulation of resistance to genotoxic stress explain the opposite outcomes observed during the overexpression of the same genes in different experiments (Table 1 and Table 2). The same opposite outcomes are observed on the level of functional groups of gene, as obtained using PANTHER classification system [70,71]. The classification shows that researchers mainly chose the genes encoding nucleic acid binding proteins and proteins that catalyze redox reactions. This is expected, since the many proteins of these groups are involved in DNA repair and oxidative stress defence, respectively. At the same time, if we divide the experiments that are based on the direction of the effect on stress-resistance, the ratio of the functional groups does not change significantly (Figure 2). This means that we cannot say that in fact overexpression of the genes of one of these functional groups increases the stress resistance more effectively than the overexpression of the genes of the other group. At the level of the whole organism, potential disruptions of functional interactions between cells, tissues, organs, and organ systems are added to the intra-cellular mechanisms of imbalance listed above. But improvements in survival, decrease in frequency of mutations, fewer incidence of cancer, and some others desirable outcomes are still observed as a result of overexpression of stress-responsive genes in a number of studies, which holds promise (Table 2).

In addition to the above, there are, apparently, many other factors that can radically change the influence of overexpression of certain genes on cellular stress-resistance. This is supported by the cell line specific effect of overexpression of the proto-oncogene *HER2*/*neu* in human breast and ovarian cancer cells. In six different cell lines, overexpression led to either a decrease, or an increase in sensitivity to chemotherapeutic agents of different classes [72]. These experimental data provide additional evidence in favor of the need for further studies of genetic regulation of stress resistance in normal and cancerous cells, as well as the stress-resistance of an organism as a whole.

## 4. Prospects

The decrease in stress-resistance of cells in the variety of experiments described above is largely caused with the multicomponent nature of stress response mechanisms that the studied genes participate in. Numerous experimental data that support the high efficiency of overexpression of the *MGMT* gene support confirm this assumption (Table 1 and Table 2). Product of this gene solely performs recognition and repair of damaged DNA bases, in contrast to most other elements of cell protective systems that operate in cooperation with many other gene products [73]. When considering the accumulated detailed knowledge of such interactions, the development of multiplex gene activation systems with mutant RNA-guided Cas9 protein opens up the widest opportunities for studying the regulation of stress resistance. Multiplex activation using one large [74] or a number of small [16] plasmids, using activators with different degrees of efficiencies, allows for selecting the appropriate range of activation. To some extent, the level of overexpression of individual genes can be adjusted by selecting sgRNA for sequences that are located at different distances from the transcription start site.

## Figures and Tables

**Table 1:** Effect of overexpression of stress responsive genes on resistance to genotoxic agents in vitro.

Gene (Gene ID *; Origin If Different)	Cells	Agents	R *	References
**Genes involved in DNA damage recognition and repair**				
*RPA3* (6119)	Human nasopharyngeal carcinoma (CNE2, HK1)	X-ray	↑	[75]
*XPA* (7507)	SV-40 transformed primary human cells	UV	↑	[76]
*APN1* (853746; yeast) coding homolog of mammalian APE1	Chinese hamster (CHO-9)	MMS	↑	[77]
		H_2_O_2_	↑	[77]
*APE1* (328)	Chinese hamster (CHO)	dioxolane cytidine	↑	[67]
	Mammalian cells	γ-ray	0	[67,78]
		alkylating agents	0	[67,68,78]
	Chinese hamster (CHO)	H_2_O_2_	0	[67]
		mitomycin C, porfiromycin, daunorubicin and aziridinyl benzoquinone (drugs that are activated by reduction)	↓	[68]
	Chinese hamster XRCC1-deficient (CHO)	alkylating agents	↓	[79]
Chimeric *MGMT* (4255) + *APE1* (328)	Human cervix adenocarcinoma (HeLa)	alkylating agents	↑	[80]
*Ku70* (2547)	Human renal carcinoma 786-O	γ-ray	↑	[81]
*Ku70* (2547; human) + *Ku80* (34930; human)	Rat cell lines Rat-1 and R708	X-ray	↓	[82]
*DNA-PK* (5591)	Human promyelocytic leukemia HL60	adriamycin	↑	[83]
*Rad51* (5888)	Mammalian cells	γ-ray	↑	[84,85]
	Chinese hamster (V79)	etoposide, hydroxyurea, thymidine	↑	[86]
	Mouse hybridoma cells	mitomycin C	↑	[85]
*Prpf19* (27339)	Human umbilical vein/vascular endothelium cells (HUVECs)	bleomycin, DL-buthionine-sulfoximine	↑	[11]
*ALC1* (9557)	Human osteosarcoma U2OS cells	phleomycin	↓	[87]
*Lig III* (3980)	Human cervix adenocarcinoma (HeLa S3)	MNNG	↑	[88]
*DNA pol β* (5423)	Chinese hamster (CHO)	cisplatin, melphalan, mechlorethamine	↑↓	[57]
	Mouse embryo fibroblast (MEF)	MMS	↑0↓	[60]
*Tag* (947137; *E. coli*) coding methyladenine DNA glycosylase I	Chinese hamster (V79)	MMS, MNU, EMS	↑	[89,90]
		MNU, ENU	0	[90]
	Murine fibroblast (NIH3T3) and murine H1 melanoma cells (B78)	MNU, MNNG, DMS, temozolomlde	0	[91]
*AlkA* (947371; *E. coli*) coding methyladenine DNA glycosylase II	Chinese hamster (V79 and Irs1)	DMS, EMS, MMS	↑	[92]
*MPG* (4350)	Chinese hamster (V79 and Irs1)	DMS, EMS, MMS	↑	[92]
	Chinese hamster (CHO)	MMS	↓	[93]
		bis-chloroethylnitrosourea, melphalan	0	[94]
		DMS, EMS, MMS	0	[95]
		MMS, MNNG	↓	[66]
	Mouse embryo fibroblast (MEF)	temozolomide	↓	[96,97]
*FPG* (946765; *E. coli*) coding homolog of mammalian OGG1	Chinese hamster (CHO and V79)	γ-ray	↑	[98]
	Chinese hamster (CHO)	aziridine	↑	[99]
*dOGG1* (31806)	Drosophila S2 cells	paraquat, H_2_O_2_	↓	[100]
		*S*-nitroso-*N*-acetylpenicillamine	↑	[100]
*OGG1* (4968; human)	Chinese hamster (AA8 and AS52)	potassium bromate or [R]-1-[(10-chloro-4-oxo-3-phenyl-4*H*-benzo[a]quinolizine-1-yl)-carbonyl]-2-pyrrolidinemethanol plus light	↑	[101]
*ERCC1* (2067; human)	Chinese hamster (AA8)	melphalan, cisplatin	↓	[102]
		UV	0	[102]
*NTH (947122*; *E.coli*)	Chinese hamster (XRS7)	γ-ray	0	[103]
		H_2_O_2_	↑	[103]
		bleomycin	↓	[103]
*Ogt* (945853; *E. coli*)	Mammalian cells	alkylating agents	↑	[104,105,106]
*Ada* (946710; *E. coli*) and its truncated and modified versions	Mammalian cells	alkylating agents	↑	[104,105,106,107,108,109,110,111,112,113,114,115,116,117]
	Chinese hamster lung fibroblasts	dibromoalkanes	↓	[105]
	Chinese hamster (V79)	MMS, HN_2_	0	[114]
	Chinese hamster (CHO)	UV, ENU	0	[112]
*MGMT* (4255) and its modified versions	Mammalian cells	alkylating agents	↑	[112,118,119,120,121,122,123,124,125]
	Chinese hamster (CHO)	UV, ENU	0	[112]
*alkB* (946708; *E. coli*)	Human cervix adenocarcinoma (HeLa)	MMS, DMS	↑	[126]
**Genes involved in detoxification and efflux of free radicals and xenobiotics**				
*SOD1* (6647)	Human lymphoblastoid cells (TK6)	γ-ray	0	[127]
	Human primary lung fibroblasts (HPLF)	γ-ray	↑	[128]
	Astrocytes of mice	xanthine oxidase with hypoxanthine, menadione	↑	[129]
	Brain neurons of mice	*S*-nitroso-*N*-acetylpenicillamine, spermine-NONOate, diethylamine-NONOate	↑	[130]
		H_2_O_2_	0	[130]
		menadione	↓	[130]
	Normal human keratinocytes	UV	0	[131]
	Human glioma cells (U118-9)	γ-ray	↑	[132]
*SOD2* (6648)	Human lung adenocarcinoma	cisplatin	↑	[133]
	Human cells	γ-ray	↑	[127,128,134,135]
	Human lymphoblastoid cells (TK6)	paraquat	↑	[127]
	Human hepatocellular carcinoma cells (HLE)	X-ray	↑	[136]
	Human gastric carcinoma cells	doxorubicin	↑	[37]
*ALDH3A1* (218)	Human adenocarcinoma cells (MCF7)	4-hydroxyperoxycyclophosphamide, doxorubicin, etoposide, 5-fluorouracil, γ-ray, H_2_O_2_	↑	[137]
*CAT* (847)	Normal human keratinocytes	UV	↑	[131]
	Mouse aortic endothelial cells (MAECs)	benzo(a)pyrene	↑	[138]
*TRX* (41737)	Drosophila S2 Cells	H_2_O_2_	↑	[139]
*MTII* (17750)	Chinese hamster ovary cells (K1-2)	Cadmium chloride, MNU, MNNG	↑	[140]
		γ-ray, bleomycin, MMS, *N*-hydroxyethyl-*N*-hloroethylnitrosourea	0	[140]
	Mouse C127	cisplatin, melphalan, chlorambucil	↑	[141]
		5-fluorouracil, vincristine	0	[141]
	Mouse β-cell	streptozotocin	↑	[129]
*MTI* (17748)	Mouse embryo fibroblasts (NIH/3T3)	tert-butyl hydroperoxide	↑	[142]
	Chinese hamster (V79)	Amsacrine, menadione, arsenite, TPA	↑	[143]
		Zn(II)	↑	[144]
		alkylating agents	0	[144]
**Genes involved in control of proliferation and cell cycle**				
*CCND1* (595)	Human adenocarcinoma cells (MCF7)	γ-ray	↓	[145]
*p21* (1026)	Glioma cells (T-98G, U-251MG with mutant p53 allele and U-87MG with wild-type p53). Medulloblastoma cells MED-3.	γ-ray	↑	[146]
**Genes involved in regulation of apoptosis**				
*BCL2* (596)	Mice thymocytes	Ionizing radiation (not specified)	↑	[147]
	Rat 6 fibroblast (R6)	UV	↑	[148]
	Human bladder cancer cells BIU87	adriamycin	↑	[149]
	Mouse embryo fibroblasts (NIH/3T3)	γ-ray	↑	[150]
	Human breast cancer cells (MDA-MB-231)	γ-ray	↑	[150]
	Human non-small cell lung carcinoma (H1299)	Ionizing radiation (not specified)	↓	[151]
**Genes with other function**				
*USP22* (23326)	Human lung carcinoma cells (A549)	cisplatin	↑	[152]
*IGF1R* (3480)	Mammalian cells	γ-ray	↑	[153,154,155,156,157]
*Sirt1* (23411)	Hepatocellular carcinoma cells (SK-Hep1)	doxorubicin	↑	[158]
	Human skin fibroblasts (HS27)	UV	↑	[159]
	Human endometrial carcinoma cells (HHUA)	cisplatin	↑	[160]
	Human gastric cancer cells (SGC7901)	adriamycin, cisplatin, fluorouracil	↑	[161]
	Normal human foreskin fibroblasts (HCA2)	Endonuclease induced DBS	0	[162]
*Sirt2* (22933)	Normal human foreskin fibroblasts (HCA2)	Endonuclease induced DBS	0	[162]
*NAMPT* (10135)	Human prostate adenocarcinoma cells (LNCaP)	H_2_O_2_	↑	[163]
*VASH1* (22846)	Human umbilical vein/vascular endothelium cells (HUVECs)	H_2_O_2_	↑	[164]
*Sirt6* (51548)	Normal human foreskin fibroblasts (HCA2)	Endonuclease induced DBS, paraquat, neocarzinostatin	↑	[162]
*Sirt7* (51547)	Mouse embryo fibroblasts (NIH/3T3)	doxorubicin	↑	[165]
	Normal human foreskin fibroblasts (HCA2)	Endonuclease induced DBS	↑	[162]
*BRCC3* (79184)	Nasopharyngeal carcinoma cells (CNE2)	X-ray	↑	[166]
*Bmi1* (12151)	Mice hematopoietic stem cells	γ-ray	0	[167]
*STAT1* (6772)	Human head and neck squamous cell carcinoma cells (SCC-61)	X-ray	↑	[45]
*SLC25A11* (67863)	Mouse motoneuron-like cells (NSC34)	H_2_O_2_, ethacrynic acid, sodium nitroprusside	↑	[168]
*ICAM-3* (3385)	Human lung carcinoma cells (H1299)	γ-ray	↑	[40]
*AKR1C3* (8644)	Human prostate cells (DU145)	6 MV photons	↑	[169]
*Pin1* (5300)	Cervix epidermoid carcinoma (Me180)	cisplatin	↑	[170]
*PVT1* (5820)	Human cancer cell lines	cisplatin	↑	[171,172]
*WRAP53* (55135)	Human osteosarcoma cells (U2OS)	γ-ray	↑	[173]
*TRF2* (7014)	Human fibroblasts (MRC-5)	H_2_O_2_	↑	[174]
	Normal human foreskin fibroblasts (HCA2)	Endonuclease induced DBS	↑	[175]
*MYC* (4609)	Normal human foreskin fibroblasts	γ-ray	↓	[176]
*TEIF* (57410)	Human cervix adenocarcinoma (HeLa)	H_2_O_2_	↑	[177]
*PARP1* (142)	Rat ovarian tumor cells (O-342)	γ-ray, MNNG	↓	[178]
		cisplatin	0	[178]
	Chinese hamster (C060)	γ-ray	↓	[179]
	Chinese hamster (CHO)	UV, MMS	↑	[180]
*HOTAIR* (100124700)	Human ovarian carcinoma cells (2780)	cisplatin	↑	[181]
*RPS3* (42761; *Drosophila*)	Human bone marrow cells from Fanconi anemia patients	mitomycin C	↑	[182]
	Drosophila S2 cells	paraquat, H_2_O_2_	↓	[100]
		*S*-nitroso-*N*-acetylpenicillamine	↑	[100]
*RPS3* (6188)	Human skin fibroblasts	UV	↑	[183]
*CAIII* (54232; rat)	Mouse embryo fibroblasts (NIH/3T3)	H_2_O_2_	↑	[184]
constitutively active *PI3K p110* (170911)	Rat embryo fibroblasts (MR4) and human papilloma cells (RT4)	γ-ray	↑	[26]
*p53* (7157)	Multidrug resistant human osteosarcoma cells (U-2OSR2 and KHOSR2)	taxol, cisplatin, doxorubicin	↓	[185]
	Human non–small cell lung cancer (A549, H1299) and colon cancer cell lines (HCT116 p53+/+, HCT116 p53−/−)	bleomycin	↓	[186]
	Human non–small cell lung cancer (A549; H1299; H358)	cisplatin, paclitaxel	↓	[187]
	Human colon cancer cells (HT29)	γ-ray	↓ 0	[188]
*SMAR1* (54971)	Human adenocarcinoma cells (MCF7)	Irradiation by ^89^SrCl2	↓	[189]

Gene ID *—EntrezGene ID for the organism from which the cDNA originated. When listed experiments performed in different species the human EntrezGene ID are specified. R *—resistance estimated based on survival, growth inhibition, DNA damage and mutagenesis andpoints. MNU—*N*-methyl-*N*-nitrosourea; ENU—*N*-ethyl-*N*-nitrosourea; MMS—methylmethanesulphonate; EMS—ethylmethanesulfonate; MNNG—*N*-methyl-*N*’-nitro-*N*-nitrosoguanidine; DMS—dimethylsulfate.

**Table 2:** Effect of overexpression of stress responsive genes on resistance to genotoxic agents in vivo.

Gene (Gene ID *; Origin, If Different)	Object	Overexpression Specificity	Agents	R *	References
**Genes involved in DNA damage recognition and repair**					
*mus210* (36697)	*D. melanogaster*	ubiquitous	γ-ray	0	[190]
			paraquat	↓	[191]
*mei9* (31373)	*D. melanogaster*	ubiquitous	γ-ray	↓	[190]
			paraquat	♂—↑; ♀—0	[191]
		neurospecific	paraquat	↓	[191]
*Rrp1* (33500)	*D. melanogaster*	ubiquitous	paraquat	♂—↑; ♀—0	[191]
			γ-ray	↓	[190]
*Ku80* (34930)	*D. melanogaster*	ubiquitous	γ-ray	0	[190]
			paraquat	♂—↑; ♀—0	[191]
*Brca2* (37916)	*D. melanogaster*	ubiquitous	γ-ray	0	[190]
*spnB* (41746)	*D. melanogaster*	ubiquitous	γ-ray	0	[190]
*dPrp19* (37123)	*D. melanogaster*	ubiquitous	paraquat, cisplatin	♀—↑	[192]
*Ada* (946710; *E. coli*) and its truncated and modified versions	*M*. *musculus*	ubiquitous	dimethylnitrosamine, diethylnitrosamine	↑	[193]
		hepatic	MNU, nitrosodimethylamine	↑	[194]
*MGMT* (4255) and its modified versions	*M*. *musculus*	bone marrow	alkylating agents	↑	[119,120,124,195]
		ubiquitous but predominantly in the thymus	alkylating agents	↑	[53,55,56,196,197,198]
		epidermal	alkylating agents	↑	[51,199]
		lung	4-(methylnitrosamino)-1-(3-pyridyl)-1-butanone	↑	[54]
**Genes involved in detoxification and efflux of free radicals and xenobiotics**					
*Gclc* (53581)	*D. melanogaster*	ubiquitous	paraquat	↑	[200]
*SOD1* (6647)	*D. melanogaster*	motorneurons	paraquat	↑	[201]
			γ-ray	↑	[201]
		ubiquitous	paraquat	0	[202]
	*M*. *musculus*	ubiquitous	benzo(a)pyrene	↑	[203,204]
*SOD2* (36878)	*D. melanogaster*	ubiquitous	100% O_2_	0	[205]
*EC-SOD* (6649)	*M*. *musculus*	alveolar type II and nonciliated distal bronchial epithelial cells	4-MV photons	↑	[206]
*CAT* (847)	*D. melanogaster*	ubiquitous	H_2_O_2_	↑	[207]
	*M*. *musculus*	heart-specific	doxorubicin	↑	[208]
		ubiquitous	benzo(a)pyrene	↑	[203,204]
			proton irradiation	↑	[209,210]
*MTII* (17750)	*M*. *musculus*	ubiquitous	streptozotocin	↑	[129]
**Genes involved in control of proliferation and cell cycle**					
*Mnk* (35288)	*D. melanogaster*	neurospecific	paraquat	↓	[191]
*dGADD45* (35646)	*D. melanogaster*	ubiquitous	γ-ray	↓	[190]
		neurospecific	paraquat	♂—↑; ♀—0	[211]
			γ-ray	0	[211]
**Genes involved in regulation of apoptosis**					
*BCL2* (596; human)	*M*. *musculus*	ubiquitous	X-ray	↑	[212]
**Genes with other function**					
*WRNexo* (42208)	*D. melanogaster*	neurospecific	paraquat	↓	[191]
		ubiquitous	γ-ray	0	[190]
*Per* (31251)	*D. melanogaster*	neurospecific	paraquat	↑	[213]
CLOCK (38872)	*D. melanogaster*	neurospecific	paraquat	↑	[213]
*Cyc* (40162)	*D. melanogaster*	neurospecific	paraquat	↓	[213]
*IGF1R*_(3480; human)	KSN nude *M*. *musculus*	tumor generated by transgenic HeLa cells	X-ray	↑	[153]
*Sirt1* (93759)	*M*. *musculus*	heart-specific	paraquat	↑	[214]
*VASH1* (22846; human)	*M*. *musculus*	intratracheally infected with adenovirus vector encoding human VASH1	paraquat	↑	[164]
*dFOXO* (41709)	*D. melanogaster*	pericerebral fat body	paraquat	↑	[215]

Gene ID *—EntrezGene ID for the organism from which the cDNA originated. When listed experiments performed in different species the human EntrezGene ID are specified. R *—resistance estimated based on survival, growth inhibition, DNA damage, mutagenesis or neoplastic transformation andpoints.
